# Caffeine Consumption and Behavioral Symptoms in Nursing Home Residents: A Cross-Sectional Analysis

**DOI:** 10.1007/s12603-020-1436-y

**Published:** 2020-07-10

**Authors:** M.A. Kromhout (Michelle), N. Rius Ottenheim, H. Putter, M.E. Numans, W.P. Achterberg

**Affiliations:** 1 Department of Public Health and Primary Care, V0-P, Leiden University Medical Centre, PO Box 9600, 2300 RC, Leiden, The Netherlands; 2 Department of Psychiatry, B1-P, Leiden University Medical Centre, PO Box 9600, 2300 RC, Leiden, The Netherlands; 3 Department of Biomedical Data Sciences, S5-P, Leiden University Medical Centre, PO Box 9600, 2300 RC, Leiden, The Netherlands

**Keywords:** Behavioral symptoms, caffeine, nursing home, dementia

## Abstract

**Objective:**

Although behavioral changes are common in nursing home residents with dementia and caffeine is known to influence behavior in healthy adults, the effects of caffeine on the behavior of persons with dementia has received little attention. In this study we assessed the relationship of caffeine and behavioral symptoms in older persons with dementia.

**Design:**

A multicenter sub-cohort study embedded in the Elderly Care Physicians (ECP) training program.

**Setting:**

Dutch nursing homes associated with the ECP training program.

**Participants:**

A total of 206 individuals with both diabetes and dementia resident in Dutch nursing homes.

**Measurements:**

Trainee ECPs collected data on caffeine consumption, cognition and behavioral symptoms using the NPI-NH, MDS-DRS and AES-C. Data on factors known to influence behavior in persons with dementia (e.g. marital status, kidney function, urinary tract infection and medication) were also collected.

**Results:**

Of the 206 participants, 70% showed behavioral symptoms. An increase in caffeine consumption was associated with a decrease in the presence of behavioral symptoms in the NPI-NH cluster affect and NPI-NH item agitation. Caffeine consumption groups also differed on the presence of disinhibition and depression. In addition, the severity of dementia influenced agitation, anxiety and the clusters affect and psychomotor.

**Conclusion:**

In a large group of older persons with dementia resident in nursing homes, a low daily consumption of caffeine was associated with greater behavioral symptoms.

## Introduction

By our definition, behavior is an ‘observable response to a particular situation'. The mechanisms underlying behavior are mostly unconscious, complex and are probably shaped by a range of factors. The consumption of coffee and other caffeinated beverages is known to influence behavior. In healthy adults, reviews show that normal caffeine consumption increases alertness and attention (1., 2., 3.), elevates mood (2), and reduces fatigue (3). At higher dosages (usually ≥ 300 mg) caffeine is known to increase anxiety (2, 3), induce psychotic or manic symptoms (2), and impair sleep (3). Effects differ between individuals and people usually adjust their caffeine consumption to minimize adverse effects.

In persons with dementia, behavioral symptoms that may include aggression, agitation, or anxiety are seen in almost all cases at some point during the disease. These symptoms are also referred to as behavioral problems, behavioral and psychological symptoms of dementia' (BPSD) or neuropsychiatric symptoms. Behavioral symptoms accompanying dementia have a negative impact on a person's quality of life (4), place a heavy burden on caregivers (5), result in a high demand for care, and are therefore often the reason for nursing home admission (6). Over half of all persons admitted to Dutch nursing homes are diagnosed with cognitive disorders or dementia, and behavioral symptoms are present in more than 80% of persons with dementia resident in nursing homes (7, 8).

A review of the limited evidence currently available on caffeine consumption in persons with dementia found inconsistent data regarding the effects of caffeine administration on neuropsychiatric symptoms: high caffeine consumption was associated with less apathy in one study; in another study coffee therapy decreased the Neuropsychiatric Inventory (NPI) total score and eliminating caffeine lowered the total NPI; in a third study caffeine consumption improved sleep in some persons and eliminating caffeine improved sleep in others (9). Current data are therefore ambiguous and inconclusive, as caffeine was reported to both induce and reduce neuropsychiatric symptoms and sleeping difficulties in individual persons with dementia. Based on caffeine research in healthy adults (1., 2., 3.) and a review in persons with dementia (9), both an increase and decrease in behavioral symptoms in persons with dementia can be expected. Specifically, we hypothesized that caffeine consumption may increase anxiety and aberrant motor behavior in persons with dementia, while decreasing apathy. Therefore, in this study we explore 1) the relation between caffeine consumption and behavioral symptoms in persons with dementia, and 2) the influence of dementia severity on any possible relationship.

## Methods

This multicenter cohort study was embedded in the Dutch Elderly Care Physician (ECP) training program, and was conducted according to a study protocol published in detail earlier.(10) Briefly, during the ECP training program, trainees spend three periods at an educational nursing home and gain experience on several types of wards (e.g. a dementia special care unit or rehabilitation unit). To allow every trainee to participate, the study population could not be limited to individuals living in a specific type of ward, which means a diagnosis of dementia could not be an inclusion criterion as this would have excluded some trainees. To create a homogenous study population that would allow inclusion of all trainees, a ward transcending inclusion measure was chosen that was not associated with behavioral symptoms in persons with dementia (10). After careful deliberation, diabetes was chosen and ECP trainees were asked to include, during their first year of training, all persons under their care who had a diagnosis of diabetes (type I or II); no other inclusion or exclusion criteria were applied. Persons were included over three consecutive years, resulting in three cohorts. Data was collected based on medical records and interviews with nursing staff. None of the instruments used placed any burden on study subjects, and the study protocol was approved by the Medical Ethics Committee of the Leiden University Medical Centre. In accordance with Dutch legislation, a waiver was given for a full review procedure by the Medical Ethics Committee because no rules of conduct were imposed and no participants were subjected to any (medical) intervention.

### Data collection

Patient characteristics (age, gender) and known factors influencing behavior in persons with dementia (e.g. marital status, kidney function, presence of a urinary tract infection and medication) were collected from the medical records.

In interviews with nursing staff, the presence of pain, the modified Barthel Index (BI), the stage of cognitive decline, caffeine consumption and behavioral symptoms were measured. The presence of pain in the last 7 days was scored using the subscale of the Minimal Data Set Resident Assessment Instrument (MDS RAI) (11).

The modified BI (12) measures mobility and dependency in activities of daily living (ADL) and records (using 10 items) the ADL of each patient. Total scores ranges from 0 (completely dependent) to 20 (complete functional independence). When used in older people, the BI has a high inter-rater reliability for the total score, and a fair to moderate agreement for the individual items (13).

The stage of cognitive function was assessed using the Reisberg Global Deterioration Scale (GDS), which consists of seven stages ranging from 1 (no cognitive decline) to 7 (very severe cognitive decline/severe dementia) (14).

To measure caffeine consumption the number of cups of coffee, tea and cola consumed was observed and recorded six times a day. The amount of caffeine in coffee differs depending on how coffee is brewed: the longer the coffee grounds are in contact with water, the more caffeine is released. Therefore, the manner of coffee preparation was also noted, and based on the brewing method, ingested caffeine was estimated in milligrams (mg) as in previous studies (15, 16) and subjects divided into three groups based on low, normal or high caffeine consumption.

Behavioral symptoms were measured using the Neuropsychiatric Inventory-Nursing Home edition (NPI-NH) (17., 18., 19.), depression was measured with the Minimum Data Set Depression Rating Scale (MDS-DRS)(20) and apathy with the Dutch Apathy Evaluation Scale-Clinician version (AES-C) (21, 22).

The NPI-NH assesses the severity and frequency of 12 different types of behavioral symptoms: delusions, hallucinations, agitation, depression/dysphoria, anxiety, euphoria/elation, apathy/indifference, disinhibition, irritability/lability, aberrant motor behavior, nighttime disturbances and appetite/eating change. For each symptom, an item score can be calculated by multiplying the severity and frequency scores. The total score is the sum of all the symptom scores. Symptom scores range from 0–12, and the total score ranges from 0–144. The NPI-NH items can also be combined in clinically meaningful clusters: cluster psychosis (delusions and hallucinations), cluster psychomotor (agitation, disinhibition and irritability) and affect (depression and euphoria) (17). The NPI-NH is a valid and reliable tool in Dutch nursing home settings and shows high inter-rater agreement (17). Individual NPI-NH items with a score of ≥ 4 are considered clinically relevant (7, 23).

The MDS-DRS is an observation-based screening instrument for depression in nursing home residents (20). It consists of seven items that are scored 0–1–2, irrespective of the assumed cause, and total scores range from 0–14. At a cut-off score of 3, the MDS-DRS has a 91% sensitivity and 69% specificity compared to the DSM-IV diagnosis of depression (20).

Apathy was measured using the 18 item AES-C (21, 22). Total scores range from 18–72 and a score ≥ 38 is indicative of apathy (21, 22). The AES-C has a high inter-rater reliability and can be used to discriminate between apathy and depression (21).

### Statistical analysis

The three cohorts were merged, checked for duplicate cases and a subgroup created containing only persons with dementia. Patient characteristics, behavioral characteristics, disease characteristics and caffeine consumption were then defined using descriptive analysis. Differences in caffeine consumption between persons with and without clinically significant behavioral symptoms were analyzed for total NPI-NH score, the MDS-DRS and the AES-C. The NPI-NH clusters and individual NPI-NH items were also analyzed if a behavioral symptom was present in at least 15% of participants. A chi-squared test was used to analyze categorical outcomes and the Cochran-Armitage test was used to analyze trends. A Pearson correlation coefficient was computed to assess the relationship between daily ingested caffeine (in mg) and the NPI-NH total score. Finally, multiple logistic regression, with robust standard error estimation adjusting for clustered design (General Estimated Equations (GEE)), was used to correct for potential confounding factors. Per outcome two models were considered: 1. Based on the variables age, gender and stage of cognitive decline; 2. The variables age, gender and stage of cognitive decline together with of any of the following variables that were significantly related to the specific outcome (the use of psychotropic medication, marital status, Barthel Index total score, the presence of pain, cohort and kidney function). A p value less than 0.05 was considered statistically significant. Statistical analyses were performed using the IBM Statistical Package for Social Sciences (SPSS) version 23, with additional analysis in R+.

## Results

The dementia subgroup consisted of 206 persons with a Reisberg GDS of ≥ 4 and a mean age of 82 years. Over half were female (59%) and three quarters were resident in a dementia special care unit. The average consumption of caffeine was within the normal range at 237mg a day, as high consumption is defined as over 300mg a day. The majority (70%) of participants had clinically relevant behavioral symptoms (defined as one or more NPI-NH item with a score of ≥ 4). Patient characteristics are presented in table 1.

**Table 1:** Patient characteristics for all persons with dementia, and for persons with dementia with and without clinically relevant behavioral symptoms

	**All**	**Behavioral symptoms**	
		**With**	**Without**
*Somatic*			
Age (mean ± SD)	82 years ± 9	82 years ± 8	81 years ± 10
Gender	(n=206)	(n=139)	(n=59)
Female	59%	61%	56%
Male	41%	39%	44%
Medication: number of prescriptions (mean + SD)	7 ± 3	7 ± 3	8 ± 3
Psychotropic medication	(n=199-202)	(n=132-135)	(n=58-59)
Antidepressant	20%	25%	14%
Antipsychotics	16%	19%	9%
Benzodiazepine	19%	24%	14%
Antiepileptic	9%	8%	14%
Kidney function			
(MDRD/GFR (mean ± SD))	62ml/min ± 23	61ml/min ± 21	63ml/min ± 23
Pain in the last 7 days	(n=206)	(n=139)	(n=59)
Yes	30%	34%	24%
No	70%	66%	76%
Caffeine consumption (median (range))	237mg/day (0–680)	230mg/day (0–595)	285mg/day (12–680)
*Functional status/activities of daily living*			
Barthel Index (median (range))	8 (1–20)	8 (1–20)	11 (1–20)
Social			
Department	(n=206)	(n=139)	(n=59)
Dementia special care unit	76%	78%	75%
Somatic department	24%	22%	25%
Marital status	(n=206)	(n=139)	(n=59)
Married	27%	28%	27%
Widowed	54%	55%	53%
Divorced	5%	4%	10%
Single	13%	14%	10%
*Psychological*			
Reisberg GDS	(n=206)	(n=139)	(n=59)
4	17%	14%	27%
5	35%	30%	46%
6	33%	37%	22%
7	16%	19%	5%

A small negative correlation was seen between the total NPI-NH score and the daily consumption of caffeine (r = -0.179, n = 142, p = 0.033*). No associations between caffeine consumption and apathy, as measured with the NPI-NH and the AES-C, were found.

An increase in caffeine consumption was associated with a decrease in the presence of clinically relevant behavioral symptoms in the NPI-NH cluster affect and the NPI-NH item agitation. A difference was also noted between caffeine consumption groups for the presence of disinhibition (measured with the NPI-NH) and depression (according to the MDS Depression Rating Scale), but without evidence of a trend. Other NPI-NH items, the NPI-NH clusters and the AES-C did not differ between caffeine consumption groups. (See table 2).

**Table 2:** Behavioral symptoms and caffeine consumption

**Behavioral symptoms**	**N**	**Caffeine consumption (%)**			**Statistics**	
		**Low**	**Normal**	**High**	**χ**^**2**^ (2)	**Trend test (p)**
NPI-NH						
Cluster psychosis	146				0.72, p = 0.697**	0.444
With		10	10	15	Fishers exact p = 0.765	
Without		90	90	86		
Cluster psychomotor	146				2.65, p = 0.266	0.245
With		59	43	47		
Without		41	58	53		
Cluster affect	145				7.45, p = 0.024*	0.008*
With		40	33	16		
Without		60	68	84		
Delusions	147				0.29, p = 0.864**	0.593
With		8	10	11	Fishers exact p = 0.938	
Without		92	90	89		
Hallucinations	146				0.60, p = 0.741**	0.449
With		4	5	47	Fishers exact p = 0.899	
Without		96	95	93		
Agitation	146				8.28, p = 0.016*	0.006*
With		47	28	22		
Without		53	73	78		
Depression/dysphoria	145				1.07, p = 0.586	0.312
With		20	15	13		
Without		80	85	87		
Anxiety	146				5.21, p = 0.074	0.054
With		24	25	9		
Without		77	75	91		
Euphoria/elation	147				1.71, p = 0.425**	0.319
With		8	2	4	Fishers exact p = 0.555	
Without		92	98	96		
Apathy/indifference	145					
With		32	30	24	0.98, p = 0.611	0.340
Without		68	70	76		
Disinhibition	146				7.73, p = 0.021*	0.233
With		28	5	18		
Without		73	95	82		
Irritability/lability	146				0.99, p = 0.609	0.375
With		35	28	27		
Without		65	73	73		
Aberrant motor behavior	146					
With		18	20	15	0.50, p = 0.779	0.665
Without		82	80	86		
Nighttime disturbances	147					
With		10	12	15	0.55, p = 0.758	0.458
Without		90	88	86		
Appetite	147					
With		14	5	11	1.99, p = 0.370**	0.652
Without		86	95	89	Fishers exact p = 0.404	
Other						
MDS-DRS	145				6.44, p = 0.040*	0.308
With		39	15	29		
Without		61	85	71		
AES-C	147				3.77, p = 0.152	0.064
With		52	66	70		
Without		48	34	30		


NPI-NH: Neuropsychiatric Inventory — nursing home edition, MDS-DRS: minimal data set — depression rating scale, AES-C: Apathy evaluation scale — clinicians edition; * significant; ** statistical assumptions were not met for chi-square due to low number of persons with the symptoms, therefore the results of a two-sided Fisher's Exact test in R+ is also given

We found no association between caffeine consumption and the total NPI-NH score when adjusted for the ‘in nursing home' clustered design (p = 0.572). However, there were percentage differences for the NPI-NH clusters psychomotor and affect, the NPI-NH items agitation, disinhibition and anxiety, and the MDS-DRS with respect to caffeine consumption (adjusted for model variables and for the clustered design). Persons with dementia and diabetes consuming high amounts of caffeine were less likely to have symptoms in the NPI-NH affect cluster and the NPI-NH item agitation compared to those consuming low amounts of caffeine. The group consuming low amounts of caffeine was less likely to show agitation than the group consuming normal amounts of caffeine. Persons consuming normal amounts of caffeine had fewer symptoms on the NPI-NH psychomotor cluster, and the NPI-NH items disinhibition and depression (measured with both the NPI-NH and the MDS), compared to those consuming low amounts of caffeine (see table 3 for model 1 and appendix A for model 2).

**Table 3:** Model 1 log regression analyses with robust SE estimation adjusting for in nursing home clustered design (General Estimated Equations)

**Behavioral symptom**	**n**		**GEE**		**Adjusted % of behavioral problems by caffeine consumption (%(CI))**		
		**Normal vs low (OR (CI))**	**High vs low (OR (CI))**		**Low**	**Normal**	**High**
NPI-NH							
Cluster Psychomotor	146	0.5 (0.2–1.0)*	0.6 (0.3–1.0)	0.040*	62 (48–73)	42 (28–57)	48 (36–59)
Cluster Affect	146	0.7 (0.3–1.5)	0.3 (0.1–0.7)*	0.025*	42 (31–55)	33 (17–55)	18 (9–32)
Agitation	146	0.3 (0.1–0.9)*	0.3 (0.2–0.5)*	0.000*	49 (38–60)	25 (13–42)	23 (17–32)
Depression	145	0.6 (0.2–1.8)	0.6 (0.2–2.1)	0.630	22 (12–37)	15 (6–32)	14 (6–31)
Anxiety	146	1.1 (0.6–2.2)	0.3 (0.1–1.2)	0.028*	23 (14–35)	25 (15–39)	10 (4–23)
Apathy	145	0.9 (0.4–1.8)	0.7 (0.3–1.8)	0.773	27 (15–43)	24 (13–40)	21 (11–36)
Disinhibition	146	0.1 (0.0–0.6)*	0.6 (0.2–2.2)	0.001*	25 (13–43)	4 (2–11)	17 (8–35)
Lability	146	0.6 (0.3–1.4)	0.7 (0.4–1.2)	0.313	34 (26–43)	25 (13–43)	25 (17–36)
Other							
AES-C	147	1.6 (0.7–3.9)	2.2 (0.8–6.4)	0.327	56 (38–73)	68 (48–83)	74 (56–86)
MDS	145	0.3 (0.1–0.7)*	0.7 (0.4–1.3)	0.015*	35 (23–50)	14 (8–23)	28 (18–40)


NPI-NH: Neuropsychiatric Inventory — nursing home edition, MDS-DRS: minimal data set — depression rating scale, AES-C: Apathy evaluation scale — clinicians edition; Variables entered in the model: caffeine consumption, Reisberg GDS, gender and age; * statistically significant (p value < 0,05); ** p value for difference in percentage of behavioral problems with respect to caffeine consumption group, adjusted for the variables entered in the model

The presence (%) of behavioral problems differed with the severity of dementia. In both the adjusted models, persons with moderately severe and severe dementia had a higher percentage of psychomotor behavior and agitation (p values 0.005, 0.043, 0.001 and 0.001, respectively). In the second model (in which the variables age, gender and stage of cognitive decline were entered, with addition of any variables significantly related to a specific outcome) behavioral symptoms in the NPI cluster affect and the NPI item anxiety were highest in persons with mild dementia (p values 0.046 and <0.000, respectively).

## Discussion

In this cross-sectional study of nursing home residents with dementia, we found a number of associations between caffeine consumption and behavioral symptoms, including consistent differences between caffeine consumption groups for behavioral symptoms in the NPI-NH cluster affect, the NPI-NH items agitation, disinhibition and depression, and depression as measured with the MDS-DRS. Persons consuming low amounts of caffeine were most likely to have behavioral symptoms. Furthermore, some behavioral symptoms differed between persons with mild, moderate, moderately severe and severe dementia.

Few studies to date have considered the role of caffeine consumption in behavioral symptoms in persons with dementia, and most previous studies have methodological flaws that preclude consistent conclusions (9). The results of the present study in accordance with some studies but contradict others. In the present group of older persons with diabetes and dementia, a higher total NPI-NH score initially correlated with lower caffeine consumption, but this association disappeared when adjusted for the clustered design and potential confounders. A Japanese study reported a significant drop in the total NPI score in a group that received coffee therapy compared to a control group (24). In a single subject trial of a patient with a high caffeine use and high total NPI score, a decrease was seen during the decaffeinated period (16). However, neither study corrected for potential confounders.

Regarding caffeine consumption and apathy in persons with dementia, both negative (15) and positive associations have been reported, with elimination of caffeine reportedly leading to a decrease in apathy (25). The present study found no association between caffeine consumption and apathy, as measured by the NPI-NH and the AES-C. In both of the earlier studies apathy was the least frequent behavioral symptom and the studies were conducted in dementia special care units. In the present study persons with dementia were included both from somatic departments and from dementia special care units, and apathy was one of the most frequently noted behavioral symptoms. In our experience, persons with dementia assigned to somatic departments are more likely to have vascular dementia than other forms of dementia. Apathy is known to be more common in persons with vascular dementia than in patients with Alzheimer's disease, possibly as a direct result of damaged subcortical circuits (26), and may therefore explain the above mentioned differences and lack of association in the present study.

Perhaps the most interesting finding in this study was that high caffeine consumption was consistently associated with lower agitation. This is in contrast to earlier studies, which found no relation between caffeine consumption and agitation in persons with dementia (15, 25). In addition, two single subject trials including individuals consuming very high amounts of caffeine found either no effect or a negative effect of caffeine on agitation (16). As the present study was cross-sectional, causality could not be determined and the identified association might be due to agitated persons spending less time consuming coffee (or other beverages) rather than caffeine itself reducing agitation in other persons with dementia. Agitation is known to increase as dementia progresses (27), and in our sample agitation was more common in persons with (moderately) severe dementia. Therefore, lower caffeine consumption could also be due to other dementia-related factors. Nevertheless, a dose-dependent effect of caffeine on aggression in animal models follows an inverted-U shaped curve, with lower aggression at very low and very high doses of caffeine (28). This finding suggests that the relationship between agitation and caffeine consumption may be more complex than simple positive or negative individual associations.

The group with normal caffeine consumption had fewer depressive symptoms compared to the group consuming low amounts of caffeine. Studies of depression and caffeine consumption are scarce, but two reviews (2002 and 2018) both suggested that moderate to intermediate consumption of caffeine is beneficial for depression in healthy adults (3, 29). The same pattern is visible in our study, as the group with high caffeine consumption had a higher level of depression than the normal caffeine consumption group. Caffeine is a known receptor antagonist of the A1 and A2a adenosine receptors (29), which are involved in cognition, motivation and emotions (amongst other effects). However, at high doses caffeine may no longer behave as an adenosine receptor antagonist (30), potentially explaining the U-shaped association between depression and caffeine consumption.

To the best of our knowledge, this investigation is the largest study of behavior, cognition, and caffeine consumption in older persons conducted to date. The cross-sectional design and embedding in the Dutch Elderly Care Physician training program made it possible to study such a large group. However, this design has two limitations that should be mentioned. Firstly, data were collected by ECP trainees in an educational setting rather than by professional researchers in a study setting. Nonetheless, data collection was supervised by a senior researcher and there is no reason to suppose that an ECP trainee would collect incorrect data. Secondly, the choice for a ‘ward transcending' inclusion criterion (diabetes) allowed all trainees to participate irrespective of department, but resulted in inclusion limited to persons with diabetes. As there is no evidence of a relationship between caffeine consumption and diabetes in persons over 65 years of age, and no known relationship between diabetes and behavioral symptoms, the results of this study are likely to be valid for nursing home residents with dementia without diabetes.

In conclusion, a low daily consumption of caffeine was associated with behavioral symptoms in a large group of nursing home residents with dementia and diabetes. To determine causality, intervention studies are warranted. Due to the highly individualized effects of caffeine on behavior, a study with individualized outcome measurements is preferred before firm recommendations can be made for specific groups. However, as caffeine consumption is an easily adaptable intervention, it can be considered as a potentially beneficial component in an individualized approach to neuropsychiatric symptoms in persons with dementia.

## Availability of data statement

The datasets generated and/or analyzed during the current study are not publicly available by order of the Medical Ethics Committee of the Leiden University Medical Center, but are available from the corresponding author on reasonable request.

## Electronic Supplementary Material

Appendix A Model 2. Log regression analyses with robust SE estimation adjusting for in nursing home clustered design (General Estimated Equations).

## References

[1] Einother SJ, Giesbrecht T (2013). Caffeine as an attention enhancer: reviewing existing assumptions. Psychopharmacology.

[2] Lara DR (2010). Caffeine, mental health, and psychiatric disorders. J Alzheimers Dis.

[3] Smith A (2002). Effects of caffeine on human behavior. Food and chemical toxicology: an international journal published for the British Industrial Biological Research Association.

[4] van de Ven-Vakhteeva J, Bor H, Wetzels RB, Koopmans RT, Zuidema SU (2013). The impact of antipsychotics and neuropsychiatric symptoms on the quality of life of people with dementia living in nursing homes. International journal of geriatric psychiatry.

[5] Borsje P, Hems MA, Lucassen PL, Bor H, Koopmans RT, Pot AM (2016). Psychological distress in informal caregivers of patients with dementia in primary care: course and determinants. Family practice.

[6] Yaffe K, Fox P, Newcomer R, Sands L, Lindquist K, Dane K (2002). Patient and caregiver characteristics and nursing home placement in patients with dementia. JAMA.

[7] Zuidema SU, Derksen E, Verhey FR, Koopmans RT (2007). Prevalence of neuropsychiatric symptoms in a large sample of Dutch nursing home patients with dementia. International journal of geriatric psychiatry.

[8] Zuidema SU, van der Meer MM, Pennings GA, Koopmans RT (2006). [Prevalence of behavioural problems in a group of demented nursing home patients]. Tijdschrift voor gerontologie en geriatrie.

[9] Kromhout MA, Rius Ottenheim N, Giltay E, Numans ME, Achterberg WP (2019). Caffeine and neuropsychiatric symptoms in patients with dementia: A systematic review. Exp Gerontol.

[10] Kromhout MA, van Eijk M, Pieper MJC, Chel VGM, Achterberg WP, Numans ME (2018). BeCaf study: caffeine and behaviour in nursing homes, a study protocol and EBM training program. Neth J Med.

[11] Fries BE, Simon SE, Morris JN, Flodstrom C, Bookstein FL (2001). Pain in U.S. nursing homes: validating a pain scale for the minimum data set. Gerontologist.

[12] Collin C, Wade DT, Davies S, Horne V (1988). The Barthel ADL Index: a reliability study. Int Disabil Stud.

[13] Sainsbury A, Seebass G, Bansal A, Young JB (2005). Reliability of the Barthel Index when used with older people. Age Ageing.

[14] Reisberg B, Ferris SH, de Leon MJ, Crook T (1982). The Global Deterioration Scale for assessment of primary degenerative dementia. Am J Psychiatry.

[15] Kromhout MA, Jongerling J, Achterberg WP (2014). Relation between caffeine and behavioral symptoms in elderly patients with dementia: an observational study. The journal of nutrition, health & aging.

[16] Kromhout MA, Numans ME, Achterberg WP (2017). Reducing behavioral symptoms in older patients with dementia by regulating caffeine consumption: Two single-subject trials. European Geriatric Medicine.

[17] Zuidema SU, Buursema AL, Gerritsen MG, Oosterwal KC, Smits MM, Koopmans RT (2011). Assessing neuropsychiatric symptoms in nursing home patients with dementia: reliability and Reliable Change Index of the Neuropsychiatric Inventory and the Cohen-Mansfield Agitation Inventory. International journal of geriatric psychiatry.

[18] Cummings JL, Mega M, Gray K, Rosenberg-Thompson S, Carusi DA, Gornbein J (1994). The Neuropsychiatric Inventory: comprehensive assessment of psychopathology in dementia. Neurology.

[19] Cummings JL (1997). The Neuropsychiatric Inventory: assessing psychopathology in dementia patients. Neurology.

[20] Burrows AB, Morris JN, Simon SE, Hirdes JP, Phillips C (2000). Development of a minimum data set-based depression rating scale for use in nursing homes. Age and ageing.

[21] Marin RS, Biedrzycki RC, Firinciogullari S (1991). Reliability and validity of the Apathy Evaluation Scale. Psychiatry Res.

[22] Lampe IK, Kahn RS, Heeren TJ (2001). Apathy, anhedonia, and psychomotor retardation in elderly psychiatric patients and healthy elderly individuals. J Geriatr Psychiatry Neurol.

[23] Margallo-Lana M, Swann A, O'Brien J, Fairbairn A, Reichelt K, Potkins D (2001). Prevalence and pharmacological management of behavioural and psychological symptoms amongst dementia sufferers living in care environments. International journal of geriatric psychiatry.

[24] Matsuda H, Konno S, Satoh M, Sai H, Fujii M, Sasaki H (2012). Coffee therapy for patients with behavioral and psychological symptoms of dementia. Geriatrics & gerontology international.

[25] de Pooter-Stijnman LMM, Vrijkotte S, Smalbrugge M (2018). Effect of caffeine on sleep and behaviour in nursing home residents with dementia. European Geriatric Medicine.

[26] Tiel C, Sudo FK, Calmon AB (2019). Neuropsychiatric symptoms and executive function impairments in Alzheimer's disease and vascular dementia: The role of subcortical circuits. Dement Neuropsychol.

[27] Kolanowski A, Boltz M, Galik E, Gitlin LN, Kales HC, Resnick B (2017). Determinants of behavioral and psychological symptoms of dementia: A scoping review of the evidence. Nurs Outlook.

[28] Wilson JF, Nugent NR, Baltes JE, Tokunaga S, Canic T, Young BW (2000). Effects of low doses of caffeine on aggressive behavior of male rats. Psychol Rep.

[29] Lopez-Cruz L, Salamone JD, Correa M (2018). Caffeine and Selective Adenosine Receptor Antagonists as New Therapeutic Tools for the Motivational Symptoms of Depression. Front Pharmacol.

[30] Fredholm BB, Yang J, Wang Y (2017). Low, but not high, dose caffeine is a readily available probe for adenosine actions. Mol Aspects Med.

