# Supplementary material for: Caffeine Consumption and Behavioral Symptoms in Nursing Home Residents: A Cross-Sectional Analysis
Source: J Nutr Health Aging. 2020 Jul 10;25(1):100–7. doi: 10.1007/s12603-020-1436-y (PMC12879125; doi:10.1007/s12603-020-1436-y)
Supplement: Supplementary file 1 — Appendix A Model 2. Log regression analyses with robust SE estimation adjusting for in nursing home clustered design (General Estimated Equations). [file mmc1.docx]

## Appendix A

Model 2. Log regression analyses with robust SE estimation adjusting for in nursing home clustered design (General Estimated Equations).

| **Behavioral symptom** | **n** | **GEE** | | | **Adjusted % of behavioral problems by caffeine consumption (%(CI))** | | |
| --- | --- | --- | --- | --- | --- | --- | --- |
|  |  | **Normal vs. low (OR (CI))** | **High vs. low (OR (CI))** | ***p***  ****** | **Low** | **Normal** | **High** |
| *NPI-NH* | | | | | | | |
| Cluster Psychomotor ^a,b,c,d,e,f^ | 140 | 0.4 (0.2-0.9)* | 0.6 (0.4-1.1) | 0.049* | 59 (38-76) | 36 (17-61) | 47 (27-67) |
| Cluster Affect ^a,b,c,d,e^ | 139 | 0.5 (0.3-1.2) | 0.2 (0.1-0.5)* | 0.002* | 47 (34-60) | 32 (16-54) | 17 (9-30) |
| Agitation ^a,b,c,d,e^ | 140 | 0.3 (0.1-0.9)* | 0.3 (0.2-0.5)* | 0.000* | 50 (37-62) | 24 (11-44) | 24 (17-32) |
| Depression ^a,b,c,d,e,f^ | 139 | 0.3 (0.1-0.8)* | 0.4 (0.1-1.8) | 0.056 | 22 (9-45) | 9 (3-21) | 10 (4-26) |
| Anxiety ^a,b,c,d,e,g^ | 134 | 1.5 (0.6-4.0) | 0.3 (0.1-1.6) | 0.089 | 18 (10-31) | 26 (13-44) | 7 (2-24) |
| Apathy ^a,b,c,d,g^ | 139 | 0.9 (0.4-1.9) | 0.8 (0.3-2.5) | 0.933 | 23 (11-42) | 21 (10-39) | 21 (10-37) |
| Lability ^a,b,c,d,h^ | 146 | 0.7 (0.3-1.7) | 0.8 (0.4-1.4) | 0.633 | 34 (25-45) | 27 (13-46) | 29 (19-40) |
| *Other* | | | | | | | |
| AES-C ^a,b,c,d,g,I,j^ | 141 | 1.7 (0.6-4.6) | 1.8 (0.4-8.0) | 0.567 | 48 (29-69) | 62 (44-76) | 63 (32-86) |
| MDS ^a,b,c,d,e^ | 140 | 0.3 (0.1-0.7)* | 0.6 (0.3-1.3) | 0.017* | 36 (21-54) | 13 (7-22) | 27 (17-39) |

NPI-NH: Neuropsychiatric Inventory – nursing home edition, MDS-DRS: minimal data set – depression rating scale, AES-C: Apathy evaluation scale – clinicians edition

The variables entered in the model are indicated with a superscript letter behind the behavioral symptom: ^a^ Caffeine consumption, ^b^ Reisberg GDS, ^c^ gender, ^d^ age, ^e^ the use of psychotropic medication, ^f^ marital status, ^g^ Barthel Index total score, ^h^ the presence of pain, ^i^ cohort, ^j^ kidney function

* statistically significant (p value < 0.05)

** p value for difference in percentage of behavioral problems with respect to caffeine consumption group, adjusted for the variables entered in the model
